# Copeptin adaptive response to SGLT2 inhibitors in patients with type 2 diabetes mellitus: The GliRACo study

**DOI:** 10.3389/fnins.2023.1098404

**Published:** 2023-03-20

**Authors:** Alessandro Maria Berton, Mirko Parasiliti-Caprino, Nunzia Prencipe, Fabio Bioletto, Chiara Lopez, Chiara Bona, Marina Caputo, Francesca Rumbolo, Federico Ponzetto, Fabio Settanni, Valentina Gasco, Giulio Mengozzi, Ezio Ghigo, Silvia Grottoli, Mauro Maccario, Andrea Silvio Benso

**Affiliations:** ^1^Division of Endocrinology, Diabetes and Metabolism, Department of Medical Sciences, University of Turin, Turin, Italy; ^2^Department of Health Sciences, University of Eastern Piedmont, Novara, Italy; ^3^Clinical Biochemistry Laboratory, Department of Laboratory Medicine, AOU Città della Salute e della Scienza di Torino, University Hospital, Turin, Italy

**Keywords:** arginine-vasopressin, sodium glucose co-transporter type 2 inhibitors, osmotic homeostasis, extracellular fluid, bioelectrical impedance vector analysis, renin-angiotensin-aldosterone system

## Abstract

**Introduction:**

In type 2 diabetes mellitus (T2DM), the antidiuretic system participates in the adaptation to osmotic diuresis further increasing urinary osmolality by reducing the electrolyte-free water clearance. Sodium glucose co-transporter type 2 inhibitors (SGLT2i) emphasize this mechanism, promoting persistent glycosuria and natriuresis, but also induce a greater reduction of interstitial fluids than traditional diuretics. The preservation of osmotic homeostasis is the main task of the antidiuretic system and, in turn, intracellular dehydration the main drive to vasopressin (AVP) secretion. Copeptin is a stable fragment of the AVP precursor co-secreted with AVP in an equimolar amount.

**Aim:**

To investigate the copeptin adaptive response to SGLT2i, as well as the induced changes in body fluid distribution in T2DM patients.

**Methods:**

The GliRACo study was a prospective, multicenter, observational research. Twenty-six consecutive adult patients with T2DM were recruited and randomly assigned to empagliflozin or dapagliflozin treatment. Copeptin, plasma renin activity, aldosterone and natriuretic peptides were evaluated at baseline (T0) and then 30 (T30) and 90 days (T90) after SGLT2i starting. Bioelectrical impedance vector analysis (BIVA) and ambulatory blood pressure monitoring were performed at T0 and T90.

**Results:**

Among endocrine biomarkers, only copeptin increased at T30, showing subsequent stability (7.5 pmol/L at T0, 9.8 pmol/L at T30, 9.5 pmol/L at T90; *p* = 0.001). BIVA recorded an overall tendency to dehydration at T90 with a stable proportion between extra- and intracellular fluid volumes. Twelve patients (46.1%) had a BIVA overhydration pattern at baseline and 7 of them (58.3%) resolved this condition at T90. Total body water content, extra and intracellular fluid changes were significantly affected by the underlying overhydration condition (*p* < 0.001), while copeptin did not.

**Conclusion:**

In patients with T2DM, SGLT2i promote the release of AVP, thus compensating for persistent osmotic diuresis. This mainly occurs because of a proportional dehydration process between intra and extracellular fluid (i.e., intracellular dehydration rather than extracellular dehydration). The extent of fluid reduction, but not the copeptin response, is affected by the patient’s baseline volume conditions.

**Clinical trial registration:**

Clinicaltrials.gov, identifier NCT03917758.

## Introduction

Copeptin, the C-terminal fragment of pre-provasopressin (CT-proAVP), represents a reliable biomarker of the activity of the antidiuretic system, being secreted by magnocellular hypothalamic neurons in equimolar amount with arginine-vasopressin (AVP) in response to osmotic, hemodynamic and stressful stimuli (Christ-Crain, 2019). The main task of AVP is to maintain osmotic homeostasis, promoting the passive reabsorption of water in the renal collecting ducts by activating the V2 receptors (V2R) located on the basal membrane of the principal cells.

It was shown that even in type 2 diabetes mellitus (T2DM) the antidiuretic system participates in the adaptation to osmotic diuresis, further increasing urinary osmolality by reducing the electrolyte-free water (EFW) clearance (Marton et al., 2021); in this context, sodium glucose co-transporter type 2 inhibitors (SGLT2i) appear to emphasize this mechanism, promoting persistent glycosuria and natriuresis.

In recent years, both empagliflozin (EMPA) and dapagliflozin (DAPA) have gained indication in the treatment not only of T2DM, but also of chronic heart failure (HF) with reduced ejection fraction and chronic kidney disease (CKD), thanks to their ability to reduce disease progression and exacerbations, together with overall cardiovascular mortality (Zinman et al., 2015; Wanner et al., 2016; McMurray et al., 2019; Wiviott et al., 2019).

The pathophysiological basis of these favorable effects is not yet fully understood, but one of the hypotheses is that SGLT2i could induce a greater reduction of interstitial fluids (ISF) than traditional diuretics, thus avoiding hypovolemia and acute kidney injury due to intravascular volume depletion (Lambers Heerspink et al., 2013; Hallow et al., 2018).

Indeed, significant losses of isotonic fluid able to reduce the effective circulating volume (ECV) cannot be immediately replaced by the water present in the interstitial space, since these two compartments share the same osmotic pressure. This process, called extracellular dehydration, leads to extensive neurohormonal activation, in turn associated with known harmful effects on the cardiovascular system when persisting for a long time (Cheuvront and Kenefick, 2014).

Conversely, hypotonic or electrolyte-free water losses are responsible for a mainly intracellular dehydration process, associated with a lower activation of the renin-angiotensin-aldosterone system (RAAS) and the sympathetic nervous system, in favor of an isolated osmotic response of the antidiuretic system (Cheuvront and Kenefick, 2014).

The aim of this proof-of-concept study was to investigate the copeptin adaptive response to persistent osmotic diuresis due to SGLT2i administration, as well as the induced changes in body fluid distribution, in T2DM.

## Materials and methods

### Design, population, observation times, and study setting

The GliRACo study was a prospective, multicenter research involving the Division of Endocrinology, Diabetes and Metabolism of the University Hospital “Città della Salute e della Scienza di Torino” in Turin, and the Division of Endocrinology of the University Hospital “Maggiore della Carità” in Novara. Both Local Ethics Committees of the two University Hospitals approved the study protocol (Turin: protocol n. D026280; Novara: protocol n. CE76/19) and the clinical research was conducted in accordance with the principles of Declaration of Helsinki.

**FIGURE 1:**
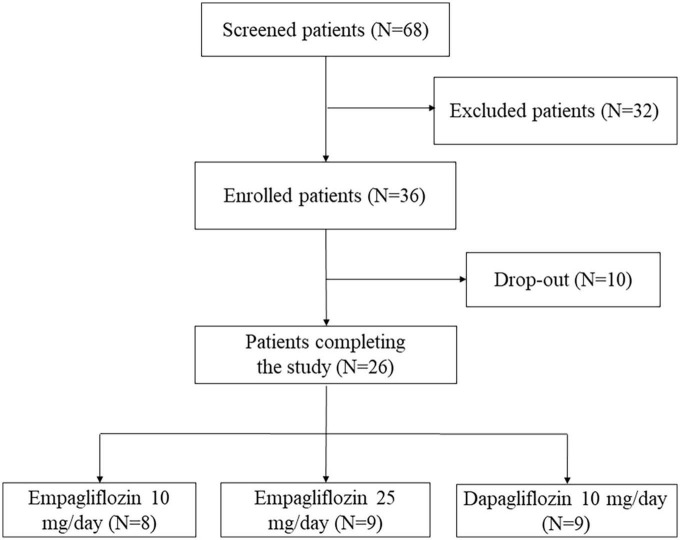
Recruitment process flow-chart.

Sixty-eight consecutive patients with T2DM (Figure 1) were evaluated at the Diabetic Outpatient Clinic of each Center for the following inclusion criteria: (1) age ≥18 years; (2) clinical indication for starting SGLT2i treatment, (3) written informed consent. Exclusion criteria were as follow: (1) past medical history positive for any disease able to alter RAAS or the antidiuretic system (i.e., primary aldosteronism, HF, CKD, liver cirrhosis, diabetes insipidus, syndrome of inappropriate antidiuresis–SIAD, adrenal insufficiency, Cushing syndrome); (2) impossibility to suspend any ongoing treatment known to alter copeptin levels or to interfere with the RAAS activity; (3) body mass index (BMI) ≥ 40 Kg/m^2^; (4) HbA1c ≥ 86 mmol/mol or clinical signs suspected of poor glycaemic control (polyuria, weight loss, visual alteration); (5) any other ongoing antihyperglycemic treatment except for metformin.

Since recruitment, any antihypertensive treatment potentially interfering with RAAS or antidiuretic system was discontinued (i.e., angiotensin converting enzyme inhibitors–ACEi, angiotensin receptor blockers - ARB, beta-blockers, diuretics) and substituted with alfa-blockers and/or calcium channel blockers. At the same time, all patients were instructed to follow controlled diet with 3–5 g of salt and up to 2 L of water per day.

Twenty days later, patients were randomly assigned to EMPA (10 or 25 mg/die, based on diabetic disease control) or DAPA (10 mg/die) alone or in addition to an ongoing metformin treatment. The day before SGLT2i starting (T0) and then at 30 (T30) and 90 days (T90) of treatment, a comprehensive fasting physical and biochemical evaluation (i.e., copeptin, plasma renin activity–PRA, aldosterone, NT-proBNP and MR-proANP) was performed; moreover, patients were instructed to avoid caffeine or nicotine on the day of study visit. In addition, at T0 and T90, all patients underwent to bioelectrical impedance vector analysis (BIVA) and 24-h ambulatory blood-pressure monitoring (ABPM).

### Analytical methods

All the biochemical analysis were performed with automated assays in the same laboratory (Clinical Biochemistry Laboratory, “Città della Salute e della Scienza” University Hospital, Turin, Italy).

As previously reported (Pasero et al., 2020), copeptin and MR-proANP concentrations (pmol/L) were determined by the B.R.A.H.A.M.S. KRYPTOR compact PLUS (Thermo Fisher Scientific, Hennigsdorf, Germany) automated method using the TRACE (Time-Resolved Amplified Cryptate Emission) technique. The detection limit of the assay was 0.9 pmol/L for copeptin and 0.05 pmol/L for MR-proANP; intra and interassay coefficients of variation were, respectively, <7 and <12% for copeptin, <4 and <11% for MR-proANP.

Plasma samples for NT-proBNP determination were processed on Cobas e602 automated platform (Roche Diagnostics) by sandwich immunoassay with two monoclonal antibodies directed against N-terminal portion (1–76) of proBNP molecule (Elecsys proBNP II), using electrochemiluminescence analysis. The limit of detection was 5 pg/mL (0.6 pmol/L), with a 5–35,000 pg/ml (0.6–4,130 pmol/L) dynamic range, as well as intra and interassay coefficients of variation < 5% at three different concentrations (46, 125 and 14, 150 pg/ml).

Serum aldosterone levels were measured by liquid chromatography coupled to mass spectrometry (LC-MS) assays. LC-MS analyses were conducted employing the MassChrom Steroids in serum/plasma kit (Chromsystems Instruments and Chemicals GmbH, Gräfelfing, Germany) on a Nexera X2 LC system (Shimadzu, Tokyo, Japan) coupled to a Triple Quad 4500MD MS (AB Sciex, Toronto, Canada). The lower limit of quantification of the method was 14 pg/ml; the intra and interassay coefficient of variation ranges from 0.9 to 1.9% and from 3.9 to 5.9%, respectively. As previously described (Parasiliti-Caprino et al., 2020), PRA (ng/ml/h) was assessed by RIA RENCTK kit (DiaSorin, Saluggia, Italy). The sensitivity of the assay was 0.20 ng/ml; the intra and interassay coefficient of variation ranges from 5.4 to 9.9% and from 7.7 to 11.5%, respectively. We decided to adopt LC-MS method for aldosterone measurement and RIA for PRA determination, because these methods have been considered the most accurate for the expected hormone range values.

Sodium fractional excretion (FENa), solute-free water (SFW) and EFW clearance have been calculated according to the following formulas: FENa = s-creatinine × u-Na/s-Na × u-creatinine × 100; SFW clearance = 24 h urine volume × [1–(u-Osm/p-Osm)]; EFW clearance = 24 h urine volume × [1–(u-Na + u-K/s-Na)].

### BIVA

Body fluid composition was evaluated by an impedance vector analyzer with measurement frequency of 50 kHz ± 1% (BIA101BIVA^®^, Akern, Loc. Montacchiello, Pisa, Italy). Both bioimpedance parameters (resistance–Rz, and reactance–Xc) were normalized according to the patients’ height (H) and plotted on a Rz/H and Xc/H graph (Biavector, Bodygram Plus^®^ version 1.31). This technique allows a reliable and reproducible assessment of the distribution of body fluids in several clinical settings (Lukaski et al., 2019), Rz reflecting conductivity through ionic solutions, Xc the impedance due to the membrane capacitance of metabolically active cells and, finally, phase angle (PhA) representing a derived parameter, which expresses the ratio between intracellular fluid (ICF) and extracellular fluid (ECF) volumes. Moreover, Biavector allows to compare the variations between repeated measurements on the same subject with the normal sex-specific ellipses of the general healthy population (Piccoli et al., 1995). Reliable thresholds for both overhydration and dehydration conditions have been previously identified at the lower and upper poles of the 75th sex-specific tolerance ellipse, respectively, (Piccoli et al., 1995).

### ABPM

Ambulatory blood-pressure monitoring were performed using an automated, non-invasive oscillometric device (TM-2430; Intermed s.r.l., Milan, Italy). Recordings were made every 15′ for the daytime and every 20′ for the night-time. Valid 24-h ABPM had to have recorded >80% of successful measurements. Controlled ambulatory BP was defined according to current guidelines (Williams et al., 2018). Heart rate (HR) variability (HRV) was determined as the standard deviation (SD) of daytime, night-time and 24-h HR. For the assessment of the nocturnal BP profile, we considered reverse dipping when night-time BP was higher than daytime, reduced dipping a night reduction of 0–10%, normal dipping a night reduction of 10–20% and extreme dipping a night reduction >20%.

### Statistical methods

Continuous variables were expressed as mean and SD or median and interquartile range (IQR) depending on their distribution; categorical variables were expressed as number and percentage. Normality was assessed using the Shapiro–Wilk test. Between-group comparisons for continuous variables were performed with the Student *t*-test or the Mann–Whitney *U*-test; repeated measures ANOVA or the Friedman test were used to identify longitudinal differences over time. Correlation between categorical variables were identified by the Chi-square test or Fisher’s exact test, as appropriate. Multivariable linear regression models with stepwise backward variable selection were adopted to assess the relationship between continuous variables during treatment with SGLT2i. With respect to BIVA, mean vectors from independent groups were compared with the two-sample Hotelling’s T2 test, while mean Biavectors’ displacement between T0 and T90 was evaluated with the paired one-sample Hotelling’s T2 test (Piccoli and Pastori, 2002).

The calculation of the sample size was based on the results reported in a recent study by Lytvyn et al. (2020), conducted in a population of 40 young type 1 diabetic subjects treated with EMPA 25 mg/die for 8 weeks, in which the authors observed a mean increase in copeptin levels equal to 1 pmol/L in normoglycemic state (i.e., 72–108 mg/dL) and 2.3 pmol/L in hyperglycemic condition (i.e., 172–198 mg/dl). Based on these data, in our analysis conducted in a real-life outpatient setting, an expected mean copeptin increase of 1.5 pmol/L in response to SGLT2i treatment was hypothesized. The expected SD was estimated to be equal to 2.2 pmol/L based on the results of the reference study (Lytvyn et al., 2020). A sample size of at least 25 patients was thus needed to obtain a statistical power of 90% (beta error 0.1) with an alpha error of 0.05.

Statistical analysis was performed using MedCalc^®^ version 20 (MedCalc Software Ltd, Ostend, Belgium).

## Results

### Study population

Among the 68 patients with T2DM evaluated to enter the study, 32 did not meet the inclusion criteria, while 36 were finally enrolled (Figure 1) and randomized to EMPA (10 mg/day in 12 patients and 25 mg/day in other 12) or DAPA (10 mg/day, 12 patients). A complete urinalysis was performed at each scheduled visit to confirm SGLT2i intake based on the presence of marked glucosuria without significant hyperglycemia. Four patients prematurely discontinued SGLT2i treatment due to symptomatic urinary infections, and six additional patients were lost at follow-up because they missed some scheduled visits; the remaining 26 patients (16 males and 10 females; age, mean ± SD, 60.9 ± 11.1 years) completed the study protocol (eight EMPA 10 mg/day, nine EMPA 25 mg/day and nine DAPA 10 mg/day).

Considering only patients who completed the study protocol, 20 were already assuming metformin [median duration of disease 6.5 (IQR 1.7–11) years]; at T0, HbA1c was ≥75 mmol/mol in three patients and fasting glucose levels were >180 mg/dl in five. Fourteen patients were already on antihypertensive treatment (mean duration of disease 9.0 ± 4.5 years), while in seven others a first diagnosis of arterial hypertension was made at T0. Among hypertensive patients, all drugs potentially interfering with the RAAS or the antidiuretic system were stopped at least 20 days before T0 (eight patients stopped ACEi or ARB, three beta-blockers and six thiazides) and an acceptable control of blood pressure levels was achieved using alpha-blockers and/or calcium channel blockers (Table 1).

**TABLE 1:** Ambulatory blood pressure monitoring (ABPM) data at baseline and at the end of the study.

ABPM variables	Baseline	90 days	*P*-value
**24 h results**			
SBP (mmHg)	134.7 ± 11.9	133.8 ± 13.4	0.577
DBP (mmHg)	78.3 ± 6.6	78.0 ± 8.7	0.684
MAP (mmHg)	96.8 ± 7.8	96.3 ± 9.6	0.625
HR (bpm)	77.7 ± 10.4	78.3 ± 8.2	0.639
HRV (bpm)	10.9 (9.1–12.3)	12.2[(10.5–13.1)	**0.011**
PP (mmHg)	56.4 ± 8.2	55.8 ± 8.8	0.566
**Day-time results**			
SBP (mmHg)	139.6 ± 13.0	136.8 ± 12.7	0.245
DBP (mmHg)	82.1 ± 7.1	80.6 ± 8.2	0.320
MAP (mmHg)	100.9 ± 8.5	99.0 ± 9.1	0.261
HR (bpm)	81.8 ± 7.7	81.5 ± 8.4	0.980
HRV (bpm)	10.3 (8.7–12.5)	11.8 (10.6–13.1)	**0.006**
PP (mmHg)	57.6 ± 9.0	56.2 ± 8.6	0.299
**Nigh-time results**			
SBP (mmHg)	124.7 ± 15.0	126.0 ± 17.0	0.901
DBP (mmHg)	70.2 ± 8.1	70.9 ± 10.7	0.974
MAP (mmHg)	88.1 ± 9.8	88.9 ± 12.1	0.948
HR (bpm)	71.9 ± 9.9	69.2 ± 9.5	**0.023**
HRV (bpm)	5 (3.7–6.9)	5 (4.3–6.2)	0.395
PP (mmHg)	54.5 ± 10.1	55.1 ± 10.6	0.919
**Nocturnal BP profile**			
Reverse dipping	4.5%	0%	0.635
Reduced dipping	54.6%	47.8%	
Normal dipping	40.9%	47.8%	
Extreme dipping	0%	4.4%	
AASI	0.5 ± 0.1	0.5 ± 0.16	0.572

Paired samples *t*-test and Wilcoxon test significant results (*p* < 0.05) reported in bold. AASI, ambulatory arterial stiffness index; BP, blood pressure; bpm, beats per minute; DBP, diastolic blood pressure; HR, heart rate; HRV, heart rate variability; MAP, mean arterial pressure; PP, pulse pressure; SBS, systolic blood pressure.

### Anthropometric and metabolic variables

An early reduction in body weight was observed at T30 (82.3 Kg at T0 vs. 81.5 Kg at T0; *p* = 0.004) with subsequent stability. Fasting glucose, but not HbA1c, resulted significantly lower at T90 (122 mg/dl at T0 vs. 115.5 mg/dl at T90; *p* = 0.041). Both absolute urinary albumin (14.9 mg/die at T0 vs. 26.7 mg/die at T90; *p* = 0.032) and urine albumin to creatinine ratio (ACR) on spot urine collection (10.5 μg/mg at T0 vs. 23.6 μg/mg at T90; *p* = 0.046) increased at T90 (Table 2).

**TABLE 2:** Changes in anthropometric, metabolic, hydro-electrolyte, and hormonal variables during the study protocol.

Variables	Baseline	30 days	90 days	*P*-value
**Anthropometric**				
Weight (Kg)	82.3 ± 21.4	81.5 ± 21.4	80.5 ± 21.1	**0.01** * ^†^
BMI (Kg/m^2^)	29.1 ± 6.0	28.8 ± 6.0	28.4 ± 5.7	**0.008** * ^†^
Waist circumference (cm)	101.5 ± 12.1	100.6 ± 11.6	100.2 ± 11.3	0.151
**Metabolic**				
HbA1c (mmol/mol)	54 (49–67)	53 (50.5–59.9)	53 (46–57)	*0.144*
P-glucose (mg/dl)	122 (113–153)	114.5 (102–144)	115.5 (104–142)	**0.038** ^†^
S-creatinine (mg/dl)	0.83 (0.73–0.91)	0.83 (0.74–0.95)	0.83 (0.71–0.91)	*0.216*
Creatinine clearance (ml/min)	110 (86–127)	105.6 (76–132)	109.5 (71–146)	*0.359*
Urinary albumin (mg/die)	14.9 (11.6–31.7)	23.2 (16.5–36.2)	26.7 (19.5–43.4)	**0.016** ^†^
ACR (μg/mg)	10.5 (7.8–25.4)	19.5 (14.1–34.2)	23.6 (14.4–33.1)	**0.027** ^†^
**Hydro-electrolyte balance**				
S-Na (mmol/L)	141 (140–142)	141 (140–142)	141 (138–142)	0.743
S-K (mmol/L)	4.5 (4.2–4.8)	4.4 (4.1–4.7)	4.5 (4.3–4.9)	0.528
P-Osm (mOsm/Kg)	291.1 ± 4.5	291.7 ± 5.4	292.1 ± 4.1	0.274
HCT (%)	42.5 (40.4–45.5)	43.2 (41.2–45.3)	46.3 (42.2–48.1)	**<0.0001** ^† ‡^
Daily diuresis (L/die)	2.0 (1.5–2.5)	2.5 (2.0–2.8)	2.1 (1.7–2.6)	***0.0006*** *
U-Osm (mOsm/Kg)	552 (510–728)	734 (637–807)	772 (697–826)	***<0.001*** * ^†^
U-Na (mmol/L)	99.6 ± 38.9	88.1 ± 23.3	89.5 ± 28.1	0.193
FENa (%)	0.76 (0.57–0.71)	0.84 (0.71–1.4)	0.89 (0.57–1.18)	***0.024*** *
EFW clearance (L/die)	0.1 ± 0.72	0.38 ± 0.45	0.28 ± 0.52	**0.042**
SFW clearance (L/die)	−1.8 (−2.7–1.5)	−3.3 (−4.5–2.5)	−3.6 (−4.5–2.6)	**<0.0001** * †
**Endocrine systems**				
Copeptin (pmol/L)	7.5 (4.7–11.9)	9.8 (7.4–16.2)	9.5 (7.4–12.5)	***0.001*** * ^†^
PRA (ng/ml/h)	0.4 (0.18–0.98)	0.66 (0.41–1.18)	0.51 (0.35–1.10)	*0.164*
Aldosterone (pg/ml)	50 (30–88)	89 (30–110)	45 (38–100)	*0.19*
ARR	112 (64.9–222.2)	69 (44.8–201.7)	94.1 (37–180)	*0.405*
NT-proBNP (pg/ml)	46 (23–77)	34 [15–67]	46.8 (16–73)	*0.33*
MR-proANP (pmol/L)	42 (36.7–79.1)	47.2 (28.5–81.5)	44.7 (30.7–72.6)	*0.16*

Repeated measures ANOVA and Friedman test significant results (*p* < 0.05) reported in bold; significant results of repeated measures ANOVA on log-normalized variables shown in italics.

*Significant difference between baseline and 30 days.

^†^Significant difference between baseline and 90 days.

^‡^Significant difference between 30 and 90 days after starting SGLT2i.

ACR, urine albumin-to-creatinine ratio; ARR, aldosterone-to-renin ratio; BMI, body mass index; bpm, beats per minute; DBP, diastolic blood pressure; EFW, electrolyte-free water; FENa, sodium fractional excretion; HbA1c, glycated hemoglobin; HCT, hematocrit; HR, heart rate; MR-proANP, mid-regional pro atrial natriuretic peptide; NT-proBNP, N-terminal prohormone of brain natriuretic peptide; p-glucose, plasma glucose; p-Osm, plasma osmolality; PRA, plasma renin activity; SBS, systolic blood pressure; s-creatinine, serum creatinine; SFW, solute-free water; s-Na, serum sodium; u-Na, urine sodium; u-Osm, urine osmolality.

### Hydro-electrolyte balance

As expected, during SGLT2i treatment u-Osm showed an early growth (522 mOsm/Kg at T0 vs. 734 mOsm/Kg at T30; *p* = 0.001), while SFW clearance further decreased (−1.8 L/die at T0 vs. −3.3 L/die at T30; *p* = 0.0002), with subsequent stability of both. Just transient increases in daily diuresis (2.0 L/die at T0 vs. 2.5 L/die at T30; *p* = 0.001) as well as in FENa were observed at T30 (0.76% at T0 vs. 0.84% at T30; *p* = 0.015), not associated to significant modification in absolute u-Na values. EFW clearance showed a slight upward trend at T30 (0.1 L/die at T0 vs. 0.38 L/die at T30; *p* = 0.044) and serum electrolytes remained stable. Of note, a significant increase in hematocrit (HCT) was recorded at T90 (42.5% at T0 vs. 46.3% at T90; *p* < 0.0001), without any significant change in p-Osm (Table 2).

### Endocrine systems

**FIGURE 2:**
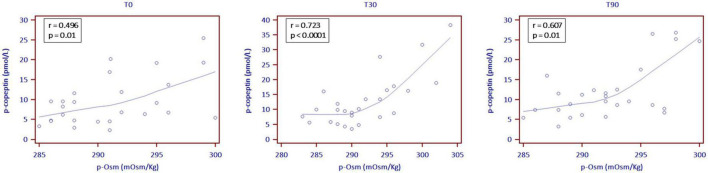
Correlation between plasma copeptin (p-copeptin) and plasma osmolality levels (p-Osm) the day before the start of SGLT2i (T0) and then at 30 (T30) and 90 (T90) treatment days (locally weighted scattered-pot smoother (LOESS) with a span of 80%).

Copeptin levels increased at T30, showing subsequent stability (7.5 pmol/L at T0, 9.8 pmol/L at T30 and 9.5 pmol/L at T90; *p* = 0.001). At all observation times p-Osm resulted a significant predictor of copeptin values (coefficient 0.06, standard error (SE) 0.03; *p* = 0.022), particularly at T30 (coefficient 0.08, SE 0.02; *p* = 0.0002) (Figure 2). Finally, at T90, copeptin was confirmed a significant predictor of log-normalized albuminuria, even if corrected for creatinine clearance or the presence of leukocyturia (coefficient 0.06, SE 0.02; *p* = 0.009). All other endocrine biomarkers remained essentially stable, in the absence of relevant clinical or biochemical signs of hypovolemia (Table 2).

### ABPM

At T90, ABPM revealed an increase in daytime HRV (10.3 bpm at T0 vs. 11.8 bpm at T90; *p* = 0.006) together with a slight decrease in mean night HR (71.9 bpm at T0 vs. 69.2 bpm at T90; *p* = 0.023). Conversely, BP did not change significantly, as reported in Table 1.

### BIVA

**FIGURE 3:**
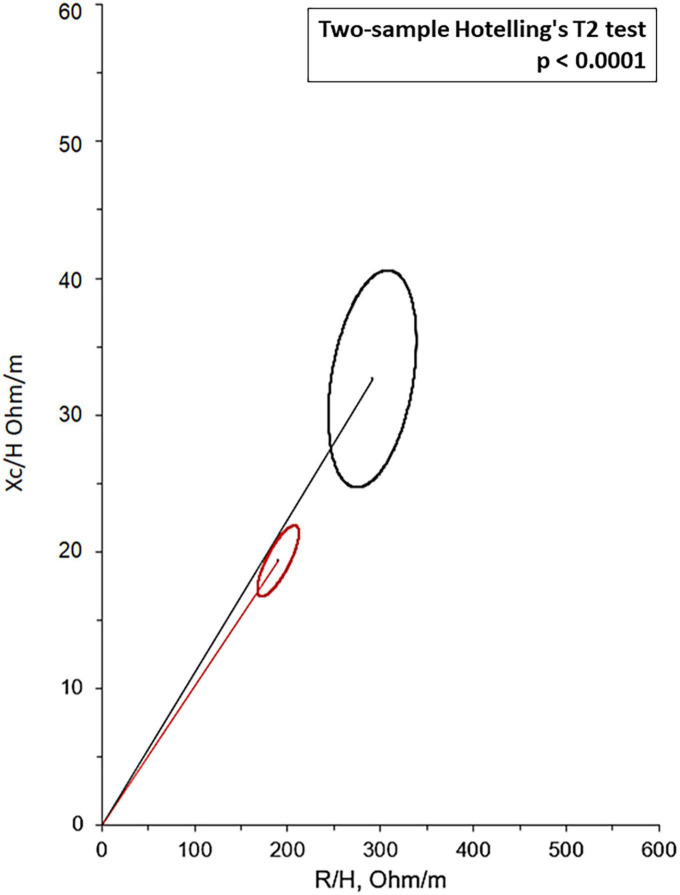
Mean Biavectors from overhydrated (red ellipse) and normohydrated patients (black ellipse), recorded the day before the start of SGLT2i (T0), compared with the two-sample Hotelling’s T2 test (*p* < 0.0001).

**FIGURE 4:**
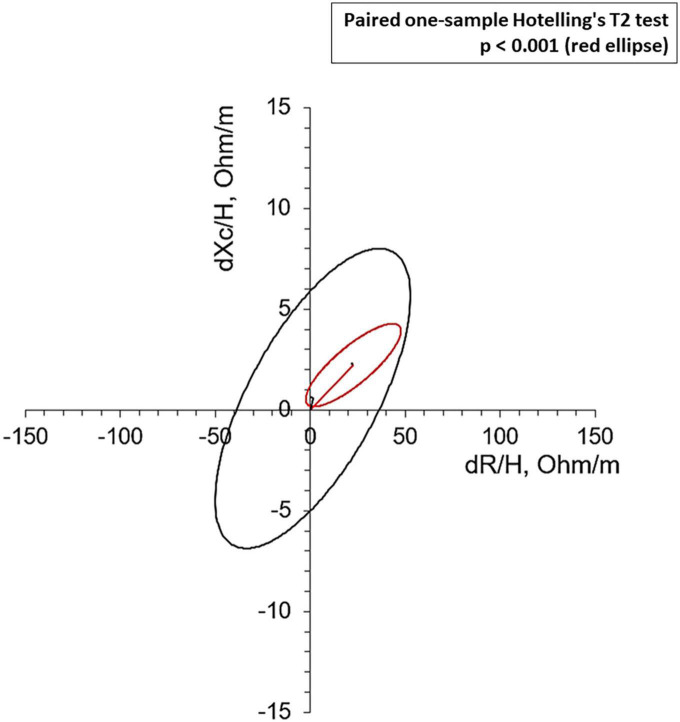
Mean Biavectors’ displacement, between the day before the start of SGLT2i (T0) and 90 treatment days (T90), from overhydrated (red ellipse, *p* < 0.001) and normohydrated patients (black ellipse, not significant), compared with the paired one-sample Hotelling’s T2 test.

Bioelectrical impedance vector analysis recorded a tendency to dehydration at T90, estimating a prevailing reduction of the ECF (22.2 L at T0 vs. 21.2 L at T90; *p* = 0.018), although in the presence of normal and stable PhA (Table 3). Twelve patients (46.1%) had a BIVA overhydration pattern (*p* < 0.0001, Figure 3) at T0 and 7 of them (58.3%) resolved this condition at T90. At the same time, in overhydrated patients both Xc and Rz increased significantly (*p* < 0.02), while ECF and total body water (TBW) decreased (*p* < 0.02). Conversely, in normohydrated diabetic patients, none of these variables changed remarkably. Finally, a significant displacement of the Biavector was confirmed at T90 only in subjects overhydrated at T0 (*p* < 0.001) (Figure 4). Repeated measures ANOVA confirmed that all volume changes (ECF, TBW, and ICF) were significantly affected by the underlying overhydration condition (*p* < 0.001), while copeptin levels and daytime HRV did not.

**TABLE 3:** Measured bioimpedance parameters and estimated body fluid composition at both baseline and end of study in all patients and, particularly, in those who achieved significant resolution of overhydration condition on Biavector.

Number of patients	Variables	Baseline	90 days	*P*-value
All patients (26, 100%)	Rz (Ohm)	388 (317−477.8)	415 (345−467)	0.354
	Xc (Ohm)	39 (33−52)	45.12 (39−54)	**0.029**
	PhA (°)	5.9 (5.5−6.4)	5.8 (5.7−6.5)	0.447
	TBW (L)	48.9 ± 11.3	47.5 ± 10.3	0.183
	ECF (L)	22.2 ± 5.2	21.2 ± 4.6	**0.018**
	ICF (L)	26.7 ± 6.7	26.3 ± 6.4	0.574
Patients with overhydration resolution (7, 27%)	Rz (Ohm)	356 (303.5−366)	410 (359.7−443.2)	**0.031**
	Xc (Ohm)	34 (33−37)	39 (37.5−43.2)	**0.016**
	PhA (°)	5.9 (5.4−6.2)	5.7 (5.3−6.4)	0.469
	TBW (L)	52.6 ± 6	47.3 ± 4.7	**0.007**
	ECF (L)	24.2 ± 2.5	22 ± 2.4	**0.002**
	ICF (L)	28.4 ± 4.1	25.2 ± 3	**0.021**

Paired samples *t*-test and Wilcoxon test significant results (*p* < 0.05) reported in bold. ECF, extracellular fluid; ICF, intracellular fluid; PhA, phase angle; Rz, resistance; Xc, reactance; TBW total body water.

### Drugs

As attended, no significant differences in endocrine and hydro-electrolyte balance variables were detected between the three different arms of treatment. No treatment was significantly associated to over-hydration resolution at T90.

## Discussion

Our results confirm an isolated response of the antidiuretic system to the administration of SGLT2i in patients with T2DM. In particular, the increase in copeptin levels seems attributable to a prevalent intracellular dehydration phenomenon, in the absence of evident signs of hypovolemia or a wide neurohormonal activation. Moreover, the dehydration process due to SGLT2i treatment led to remarkable changes in body fluid distribution only in basally overhydrated patients.

SGLT2i represent new euglycemic drugs able to induce sustained glycosuria by the inhibition of glucose reabsorption in the renal proximal tubule. SGLT2i proved to be effective in reducing cardiovascular risk in patients with T2DM beyond glycemic control, even preventing HF exacerbation and CKD progression (Zinman et al., 2015; Wanner et al., 2016; Neal et al., 2017; Wiviott et al., 2019). Moreover, regardless of T2DM, SGLT2i treatment seems able to improve all hydro-retention states such as HF, liver cirrhosis, CKD and even the syndrome of inappropriate antidiuresis (Saffo and Taddei, 2018; McMurray et al., 2019; Refardt et al., 2020; Bioletto et al., 2023). So far, several hypotheses have been formulated to explain at least some of the unexpected pleiotropic effects associated with the administration of SGLT2i, some authors arguing that these drugs can allow effective drainage of interstitial fluid (ISF), thus avoiding hypovolemia and acute kidney injury (Lambers Heerspink et al., 2013; Hallow et al., 2018).

Indeed, consistent losses of body water and electrolytes, as observed during secretory diarrhea or loop diuretics administration, may be responsible for large ECF volume contraction, primarily involving the intravascular volume. In similar conditions of marked extracellular dehydration, the activation of baroreceptors determines a reduction in the tonic inhibition exerted on the release of AVP (Cheuvront and Kenefick, 2014). Furthermore, the baroreceptor reflex itself, together with the strong stimulation of AVP 1a receptors (V1aR) by the hypothalamic nonapeptide, induces the activation of the sympathetic nervous system and the RAAS, aiming to restore the ECV both through arterial vasoconstriction and through the reabsorption of sodium (Na^+^) and water from the kidney. Unfortunately, a similar persistent neurohormonal activation also leads to harmful cardiovascular effects and cardiac remodeling, at least in part attributable to chronic water retention and the combined profibrotic action exerted by AVP, angiotensin II and aldosterone (Szczepanska-Sadowska et al., 2018).

Conversely, intracellular dehydration occurs during prevailing losses of electrolyte-free water. This condition produces a consequent flow of water from the ICF to the ECF and is detected by both the central and peripheral osmoreceptors, in turn responsible for an isolated stimulus to the release of AVP from the hypothalamus (Cheuvront and Kenefick, 2014).

Copeptin is co-secreted by the neurohypophysis in an equimolar proportion with AVP and in response to the same stimuli, being mainly osmoregulated. Due to low pre-analytical variability and reliable automated testing, copeptin levels are strongly correlated with p-Osm in healthy subjects, even better than AVP itself (Balanescu et al., 2011).

Copeptin levels were found slightly higher in patients with both type 1 and T2DM than in healthy individuals (Roussel et al., 2017; Jensen et al., 2019) and, in this regard, an acceleration in the turnover of body fluids due to glycosuria, as well as a reset of the osmostat have been hypothesized (Marton et al., 2021). Nevertheless, a definitive justification for this endocrine adaptation has not yet been provided (Bankir et al., 2001).

Furthermore, a modest increase in copeptin was previously observed even after a few weeks of treatment with SGLT2i (Heerspink et al., 2019; Lytvyn et al., 2020); thus, further supporting the theory of a compensatory mechanism to limit volume depletion in response to osmotic diuresis, but also of a possible adaptive mechanism alteration of the AVP-renal axis.

In this context, our results show for the first time that copeptin adaptation to SGLT2i in diabetic patients persists for over 3 months and that its levels remain strongly associated with p-Osm even during long-term treatment. Most importantly, BIVA recorded a proportional dehydration process between ECF and ICF; this phenomenon was associated with a condition of basal hyperhydration, in the absence of significant RAAS activation.

These data support the hypothesis of a progressive and coupled reduction of both ECF and ICF, as observed in the process of intracellular dehydration, able to counteract ECF overload without inducing hypovolemia.

Indeed, our results confirm that treatment with SGLT2i in well-controlled diabetic subjects increases u-Osm inducing glycosuria, with only a transient increase in total daily diuresis and natriuresis.

A possible explanation for these findings is the unique mechanism of action of SGLT2i. As known, the natriuretic effect of SGLT2i is exerted by the selective inhibition of Na^+^ reabsorption in the proximal tubule, with consequent increase of the luminal Na^+^ content in the distal convoluted tubule and in the collecting duct. Increased luminal Na^+^ and flow rate result in increased Na^+^ reabsorption in the aldosterone-sensitive distal nephron *via* epithelial Na^+^ channels (ENaC), thereby reducing effective Na^+^ losses (Szczepanska-Sadowska et al., 2018). Finally, it should be considered that an increase in AVP release would further improve both ENaC and Na^+^/K^+^-ATPase activity in the kidney (Nicco et al., 2001; Mordasini et al., 2005).

On the other hand, in physiological conditions (i.e., euglycemic state), serum glucose substantially represents an ineffective osmole, due to its presence both in the extracellular and intracellular compartment (Verbalis, 2003); conversely, in severe hyperglycemic state, glucose significantly increases effective p-Osm by attracting water from the ICF.

**FIGURE 5:**
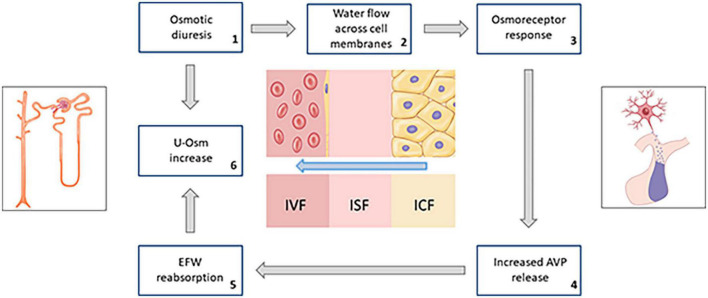
Schematic summary of the hypothesized response of osmoreceptors to water flow across cell membranes due to persistent osmotic diuresis during treatment with SGLT2i (AVP, arginine-vasopressin; EFW, electrolyte-free water; u-Osm, urine osmolality).

The result of the sum of these combined effects is that, in a population of diabetic subjects not severely hyperglycemic, but affected by ECF overload, SGLT2i would favor a progressive loss of substantially hypotonic fluids compared to plasma; thus, determining a prevalent phenomenon of intracellular dehydration (Figure 5).

Noteworthy, our results also confirm an evident increase in HCT a few months after treatment with SGLT2i. Although HTC levels are reliable indicators of a prevalent extracellular dehydration phenomenon, several evidence gathered in recent years supports the hypothesis that this new class of drugs directly and indirectly improves erythropoietin production (Marathias et al., 2020; Kanbay et al., 2022).

We also found a significant increase in HRV in our population, accompanied by a reduction in mean nocturnal HR (Table 2), apparently not associated with resolution of fluid overload, as well as copeptin response.

Consistently, a recent Japanese study identified during SGLT2i administration a significant reduction in HR at rest, another well-known indicator of the autonomic nervous system activity (Sano, 2018). Taken together, these observations could suggest a beneficial reduction in sympathetic tone and an improvement in the function of the autonomic nervous system, notoriously impaired in diabetic subjects; although this constitutes a research area yet to be explored (Spallone et al., 2011). Conversely, ABPM did not show significant blood pressure variation during SGLT2i treatment, except for small reduction in diurnal values, of the order of 2–3 mmHg, in agreement with the results of other groups (Baker et al., 2017).

Finally, recent evidence, collected from large observational studies conducted on diabetic patients, has shown a clear association between the values of copeptin and albuminuria (Velho et al., 2013; Roussel et al., 2017). In our population, administration of SGLT2i led to a subclinical increase in both albuminuria and ACR, regardless of any sign of lower urinary tract infection (Table 1). Although there is evidence of a possible adverse effect of elevated circulating AVP levels on the degree of albuminuria, possibly mediated by V2R activation, a clear pathophysiological explanation for this phenomenon has not yet been provided (Bankir et al., 2001; Roussel et al., 2017). Most of all our patients were no longer receiving RAAS inhibitors at least 3 weeks prior to the initiation of SGLT2i and this could represent an explanation for a partially increased glomerular filtration pressure.

No variables among copeptin, HVR, TBW, ECF, and ICF were significantly associated with the arms of treatment in our population. Although the number of subjects required for our study was calculated to find a significant difference in copeptin levels before and after SGLT2i treatment, these data support the hypothesis of a class effect; furthermore, a low dose of EMPA may be sufficient to achieve the same beneficial draining effect of ECF, as hypothesized based on other previous studies (Zinman et al., 2015).

The main strengths of our study are the prospective design, the outpatient population enrolled in real life and the extensive analysis conducted on the endocrine regulation of water and electrolyte balance. On the other hand, the absence of a control group and the small sample size certainly represent the main limitations of this research. Furthermore, there is a complex relationship between AVP and glucose metabolism, as V1aR activation induces both glycogenolysis and gluconeogenesis in the liver (Whitton et al., 1978; Keppens and de Wulf, 1979) and diabetic patients also exhibit an apparent enhanced AVP response to various stimuli (Bankir et al., 2001). In addition, previous studies have shown that AVP increases the release of insulin and glucagon by acting directly on pancreatic beta and alpha cells, in a glucose-dependent manner (Abu-Basha et al., 2002); whereas insulin may be able to reduce urinary Na^+^ excretion, due to its ability to promote the adenylate cyclase system in the thick ascending limb of the nephron (Mandon et al., 1993).

Also for this reason we enrolled patients in good glycemic control to evaluate the copeptin response to SGLT2i; on the other hand this implies that our results are not applicable to a poorly controlled diabetic patient population.

## Conclusion

In patients with T2DM, SGLT2i induces the release of AVP, thus compensating for persistent osmotic diuresis. This mainly occurs because of a proportional dehydration process between ECF and ICF (i.e., intracellular dehydration rather than extracellular dehydration). The extent of fluid reduction, but not the copeptin response, is affected by the patient’s baseline volume conditions. In view of the known association between T2DM, essential arterial hypertension and volume overload, even in the absence of HF, our results offer some new insight into the pleiotropic benefits derived from SGLT2i treatment in patients with T2DM.

## Data availability statement

The original contributions presented in this study are included in the article/supplementary material, further inquiries can be directed to the corresponding authors.

## Ethics statement

The studies involving human participants were reviewed and approved by the Local Ethics Committees of the Turin and Novara University Hospitals (Turin: protocol *n*. D026280; Novara: protocol *n*. CE76/19). The patients/participants provided their written informed consent to participate in this study.

## Author contributions

AMB and MP-C conceived and designed the study. FR, FP, and FS performed the biochemical analysis and collected the results. CL and CB performed the instrumental evaluation and edit the cases report form. AMB, MP-C, NP, MC, and FB performed the data analysis, the figures designing, and the manuscript writing. VG, GM were in charge for overall direction and planning. SG, MM, and ASB gave the needed encouragement and support to investigate and supervised the findings of this work. All authors revised the manuscript for important intellectual content and approved the definitive version.
